# Correlation between Breastfeeding, Maternal Body Mass Index, and Childhood Obesity

**DOI:** 10.3390/epidemiologia5030030

**Published:** 2024-07-19

**Authors:** Ermioni Palaska, Evangelia Antoniou, Maria Tzitiridou-Chatzopoulou, Panagiotis Eskitzis, Eirini Orovou

**Affiliations:** 1 Department of Midwifery, University of West Attica, 12243 Athens, Greece; lilanton@uniwa.gr (E.A.); eorovou@uniwa.gr (E.O.); 2 Department of Midwifery, University of Western Macedonia, 50200 Ptolemaida, Greece; mtzitiridou@uowm.gr (M.T.-C.); peskitzis@uowm.gr (P.E.)

**Keywords:** breastfeeding, breastfeeding duration, childhood obesity, maternal BMI, child’s BMI

## Abstract

Breast milk is a unique and highly beneficial source of nutrition for infants. It contains a wide range of nutrients that are specifically tailored to meet the specific needs of a growing infant. On the other hand, obesity is a major health issue that affects people around the world. The aim of this study is to investigate the relationship between breastfeeding and child BMI and the role of maternal BMI, which may influence this relationship. This study revealed that a longer duration of exclusive breastfeeding was associated with a decrease in the prevalence of overweight children. Additionally, the research found that children born to overweight or obese women had a higher probability of being overweight or obese themselves. Considering that mothers with a higher pre-pregnancy body weight are more likely to have children with a higher BMI, it is important that they receive information about the advantages of breastfeeding for a minimum of 6 months for both themselves and their children. Additionally, they should be offered ongoing support, to encourage them to start breastfeeding and continue with it beyond this period.

## 1. Introduction

Breastfeeding is the best way to feed neonates and infants. Breastfeeding, starting from the first hour after birth, exclusively for six months and continuing until two years or longer, is one of the most powerful practices for promoting the survival and well-being of children. Breastfeeding reduces the rates of childhood obesity, supports healthy brain development, and is associated with a higher performance in intelligence tests among children and adolescents at all income levels [1].

The nutrients present in breast milk are uniquely designed to meet the specific nutritional needs of infants and are highly bioavailable, meaning they can be easily digested and absorbed by the infant’s immature digestive system [2]. Breast milk provides a perfect balance of carbohydrates, proteins, fats, vitamins, minerals, and antibodies, which are essential for the optimal growth and development of the infant [3]. Breast milk also has a lower protein concentration compared to formula, which leads to a slower weight gain pattern [4]. Therefore, an increased intake of protein in infant formula can lead to higher concentrations of insulin and insulin-like growth factor 1 (IGF-1). This, in turn, can stimulate accelerated growth and the development of adipose tissue [5]. In previous studies, researchers have examined the impact of breast milk hormones and macronutrients on short-term infant growth and body composition [6,7]. However, these studies have not explored the potential link between these factors and the long-term risk of obesity in children. In a systematic review [8], it was observed that formula-fed infants in the first week of life have a higher energy intake and protein intake compared to breastfed infants. The energy intake of formula-fed infants was found to be 1.2 to 9.5 times higher, while their protein intake was 1.2 to 4.8 times higher, compared to breastfed infants.

Over the past decade, multiple research studies have suggested a link between breastfeeding and the risk of childhood obesity. A study conducted by the World Health Organization (WHO) revealed that extending the duration of breastfeeding can decrease the likelihood of obesity in children [9]. This finding is supported by various research studies [10,11,12]. However, it is worth noting that earlier studies conducted in American and European populations did not find a significant correlation between breastfeeding and childhood overweight and obesity [3,13], a fact that shows that the research in this area must continue.

Obesity is a significant public health problem at an international level. The risk of poor health is strongly associated with the body mass index (BMI) of a person [14]. Rates of adult obesity have increased significantly over the past four decades, nearly tripling in prevalence. Currently, it is estimated that around 13% of the global population is affected by obesity. Moreover, the risk of obesity is higher among women compared to men [9]. However, adult obesity is associated with an increased risk of various health conditions, including heart disease, stroke, certain types of cancer, and osteoarthritis. On the other hand, there is a significant burden of overweight or obesity among children. Approximately 41 million children between the ages of 0 and 5, and over 340 million children between the ages of 5 and 19, are affected by overweight or obesity [15]. Childhood obesity is a serious public health issue, because it often tracks into adulthood, increasing the risk of obesity and related health problems later in life [16,17]. Therefore, obese children are more likely to become obese adults, and this cycle can be difficult to break. Obese children are also more likely to experience victimization through bullying. They may be targeted by their peers due to their weight, leading to emotional distress and social isolation. The experience of bullying can further contribute to low self-esteem and mental health issues. Moreover, there is a social stigma associated with obesity that affects children and adults alike. Obese individuals often face discrimination, prejudice, and negative stereotypes. This stigma can have far-reaching consequences, impacting various aspects of life, including employment opportunities, relationships, and overall quality of life [18].

Childhood obesity is related to various factors, including genes, socioeconomic status, race, ethnicity, gender, lifestyle, and diet. It is a multifactorial outcome, meaning that it is related to a combination of these factors rather than a single cause [19]. Some maternal factors, such as the mother’s age, education level, family income, and maternal Body Mass Index (BMI) [19,20], have also been linked to childhood obesity. It is a fact that a mother’s high BMI affects the child’s BMI, not only due to genetic predisposition but also through the family environment. It seems that the formation of the child’s dietary behavior is significantly influenced by the habits and practices applied within the family [21]. However, early fetal exposure in the womb to insulin resistance and diabetes has been associated with various metabolic disorders in the child, as well as a high BMI [22]. A study conducted by Heerman et al. [23] utilized a large retrospective cohort and included a diverse sample. The findings revealed that pre-pregnancy obesity, when combined with excessive gestational weight gain, heightened the risk of childhood obesity during the first year of life.

Therefore, due to controversial research results and the significant effect of confounding factors on the relationship between breastfeeding and childhood obesity, we conducted a study to investigate the relationship between breastfeeding and child BMI and the role of maternal BMI, which may influence this relationship.

## 2. Materials and Methods

This specific study was carried out between July 2018 and June 2019 in daycare centers in 4 municipalities of South Athens, Greece, with a cross-sectional design. Our initial sample consisted of 674 mother–child couples. The study was approved by the Department of Nursing at the University of the Peloponnese, as well as the municipal daycare centers in southern Athens where the present research was conducted, and the relevant approvals were obtained. The parents were informed by the researcher about the purpose of the study and the importance of their participation. They were also informed about anonymity and confidentiality, as well as their right to withdraw from the study at any time. Then, a specific questionnaire was given to the parents at the daycare center after they gave their consent. The data were recorded by the parents and returned to the researcher after a week.

### 2.1. Participation Criteria

The mothers who participated in the study were required to know the Greek language and have an appropriate level of knowledge, in order to understand the purpose of the study and respond to the measure questions. The ages of the children attending the day care centers ranged from approximately 2 to 5 years old.

The only exclusion criterion was the inability to understand the Greek language. Therefore, all mothers who knew Greek and had children in daycare could participate in the study. In this particular study, all the mothers were proficient in Greek.

### 2.2. Measure

The Questionnaire for Assessing the Impact of Breastfeeding and Other Factors on the Prevention of Obesity

The questionnaire was designed specifically for the needs of the study and included three sections: (a) socio-demographic and anthropometric data, (b) information about pregnancy and childbirth, and (c) information about breastfeeding. In this specific study, content validity was used for the standardization of the questionnaire, and the test–retest method was employed to check reliability. Spearman’s rho non-parametric test was used for correlation. For the questions regarding pregnancy and childbirth, a statistically significant positive correlation was found between the first and second measurements (rho = 0.751, *p* < 0.001). Additionally, for the breastfeeding questions, a statistically significant positive correlation was found between the first and second measurements (rho = 0.900, *p* < 0.001). Based on these results, the questionnaire is considered reliable, as it demonstrated a high level of positive correlation between the two measurements.

The questionnaire aimed to gather information about the habits of mothers during the preconception and peripartum stages, which are predictive factors for childhood obesity. The questionnaire was constructed by the researcher and followed all the legal procedures for research. The questionnaire consisted of 4 parts: (a) demographic information about the parents, (b) demographic information about the children, (c) information on the pregnancy and childbirth, and (d) information on breastfeeding.

To assess the children’s BMI, we used WHO models. The data were recorded by the parents after being informed of these. The height, weight, and age were recorded, and using specific WHO charts, the Z-score and percentile ranks for the children were determined. In more detail, the WHO has developed models to describe the normal development of children from birth to 5 years under optimal environmental conditions [24]. Although these are not ideal for the entire pediatric population, these standards have been applied everywhere, regardless of nationality, socioeconomic status, and type of diet. Body Mass Index (BMI)-for-age Z-scores ≤ 0, >1.0, >2.0, and >3.0 are recommended by the WHO to classify children aged 0–5 years as at risk of being overweight, overweight, and obese, respectively. Z-scores are on a linear scale; with the same interval between distribution values, this allows the calculation of the average and standard deviation. To perform the test, it was necessary to find the Z-scores of the variables concerning the BMI for the first and second year of life. Based on these values, the following categorization was made: for values below −2, the children were categorized as cachectic; from −2 to 1, they were categorized as having a normal weight; from 1 to 2, they were categorized as having a risk of being overweight; from 2 to 3, they were categorized as overweight; and for more than 3, they were categorized as obese [25].

### 2.3. Statistical Analysis

The chi-square test for independence was used for the statistical analysis. The Fisher statistical function was used, and in addition, a Monte Carlo simulation was employed to increase the reliability of the test when the assumptions governing it did not hold (no cells with zero frequency and, at most, 20% of cells with values below 5). To perform the test, the Z-scores of variables related to the Body Mass Index (BMI) for the first and second year of life and the current year needed to be calculated.

## 3. Results

Data from 674 mother–child couples were analyzed. Fifty-six percent (50.6%) of the children were boys and 49.4% were girls. The response rate was 674/1500 = 44.93%. Additionally, the percentage of missing values ranged from 3 to 136 observations (0.4–20.2%).

The mean age of the children was 3.39 (SD = 1.01) years. The youngest age was 2.3 years and the oldest was 5.5 years. The mean age of the mothers was 36.91 years (SD = 4.50). The youngest age was 24 years and the oldest was 56 years (Table 1).

Regarding the duration of breastfeeding, only 8.5% breastfed for over 24 months, 6% breastfed for 18–24 months, 13.4% breastfed for 12–18 months, 31.9% breastfed for 6–12 months and 40.2% of mothers breastfed for 1–6 months (almost half of them (120) breastfed only for the first month).

**Figure 1:**
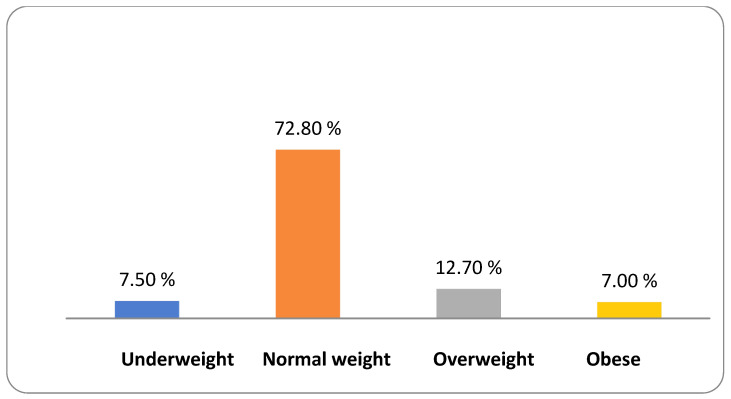
Classification of women based on BMI before pregnancy.

Based on the BMI before pregnancy, 72.8% of the mothers had a normal weight, 12.7% of the mothers were overweight, 7.5% of the women were obese, and 7% of the women were morbidly obese (Figure 1).

Based on the Z-score in the first year of the children’s lives, 84.4% had a normal weight, 11.8% were on the verge of becoming overweight, 2% were overweight, 1.2% were underweight, and 0.7% were obese (Table 2). In the second year of their lives, 81.8% of children had a normal weight, 13.7% were on the verge of becoming overweight, 3% were overweight, 1.1% were underweight, and 0.5% were obese (Table 3).

According to Table 4, statistically significant correlations were found in the following cases. For women with a normal weight, it was observed that 84.8% of boys who were breastfed for less than 6 months had a normal weight, compared to 92.2% of boys who were breastfed for over 6 months. Additionally, 1.4% of boys who were breastfed for over 6 months were overweight or obese, compared to 7.6% of boys who were breastfed for less than 6 months (Fisher’s exact test = 6.098, *p* = 0.035, CI(99%): 0.030–0.039).

For women who were overweight or obese, it was noted that 68% of boys who were breastfed for less than 6 months had a normal weight, compared to 86.0% of boys who were breastfed for over 6 months. Additionally, 12% of boys who were breastfed for less than 6 months were overweight, compared to 2.3% of boys who were breastfed for over 6 months (Fisher’s exact test = 8.312, *p* = 0.018, CI(99%): 0.015–0.022).

## 4. Discussion

The aim of this study was to investigate the relationship between breastfeeding and child BMI and the role of maternal BMI, which may influence this relationship.

An important finding of several studies was that exclusive breastfeeding and the duration of breastfeeding have a positive correlation with childhood obesity. A study by Chen et al. [26] showed that exclusive breastfeeding has a preventive effect on childhood obesity. In a national sample of 700,000 children, it was observed that those who were exclusively breastfed for a period of 4 to 6 months had a 22% lower risk of being overweight, while children who were not breastfed exclusively had a 4% lower risk of being overweight compared to those who were not breastfed at all. Furthermore, studies by Jwa [27] and Armstrong and Reilly [10] showed a decrease in the BMI of children who were exclusively or partially breastfed compared to children who were formula-fed. Children who breastfed for more than 6 months had a lower percentage of overweight and obesity compared to children who breastfed for less than 6 months. The results of our study are in agreement with a previous study [28], which showed that increasing the duration of exclusive breastfeeding reduced the percentage of overweight children. Similarly, in a study conducted by von Kries et al. in 1999 [29] in Germany, a clear effect of breastfeeding duration on the increase in children who were overweight and obese at the time of school entry was found. According to another recent study, breastfeeding can significantly reduce the prevalence of overweight and obesity among children and adolescents aged 6 to 16 years [2]. A relevant result was observed in the present study; based on the Z-score distribution, it was found that there is a statistically significant relationship between the duration of breastfeeding and a child’s ΒΜΙ between two and five years old.

The Liese et al. [28] study found that an increased duration of exclusive breastfeeding reduced the percentage of overweight children. The prevalence of overweight children was significantly higher in those who had never been breastfed compared to those who had been breastfed.

The children of overweight or obese women who became pregnant, according to the findings of this study, have a higher likelihood of being overweight or obese. Our results are consistent with those of a recent study [30], which reported that maternal obesity and overweight before pregnancy are associated with high rates of overweight and obesity in their offspring aged 2–5 years. Research conducted by Mourtakos et al. [31] yielded similar results, as it found a significant correlation between the mother’s pre-pregnancy weight and obesity among her descendants, even at the age of 8. Furthermore, Leonard et al.’s research [32] suggests that the high weight of the mother during the reproductive period increases the risk of obesity in her offspring. However, the study emphasizes that a high pre-pregnancy BMI is a stronger predictive factor for childhood obesity than weight gain during pregnancy. According to the article by Gul et al. [33], which refers to the immediate effects of maternal nutrition on the child, the pre-pregnancy nutritional status of women is an important indicator of birth weight. The nutritional status of the mother affects the development of the embryo in terms of weight, occipital–frontal circumference, and length. However, in their study, Simko et al. [34] found that the risk of having a large neonate is higher in overweight and obese mothers compared to undernourished mothers. In addition, according to Moussa et al. [35], the percentage of large-sized neonates increases linearly with the increase in maternal BMI.

Furthermore, according to our results, a statistically significant relationship was found between the gender of the child and obesity. More specifically, the results of this study indicate that boys who were breastfed for more than 6 months had a lower BMI compared to boys who were breastfed for less than 6 months from all mothers except the cachectic ones. Regarding girls, the corresponding statistical relationship was not found to be significant. However, the percentage of normal weight in girls seems to be higher in those who were breastfed for more than 6 months in all mothers except the cachectic ones. However, differences in the prevalence of obesity may be attributed to influences related to parental dietary practices [36] and socio-cultural factors [37] as well as influences related to body composition and hormones [38].

The major strength of this study is that it is the first conducted in Greece to investigate the impact of breastfeeding on childhood obesity, as well as the influence of maternal BMI on the child’s BMI. However, a limitation of the study is that the sample was convenience-based, so the results may not be generalizable. Therefore, it is advisable to conduct more studies in representative populations.

## 5. Conclusions

In conclusion, breastfeeding provides a protective effect in preventing childhood obesity, especially when extended beyond the first 6 months. Considering that mothers with a higher pre-pregnancy body weight are more likely to have children with a higher BMI, it is imperative that they be informed about the benefits of breastfeeding for at least 6 months for both themselves and their children, and also to provide continuous support to help mothers initiate and continue breastfeeding beyond 6 months.

We recommend more cohort studies with representative populations to further investigate the additional factors responsible for childhood obesity.

## Figures and Tables

**Table 1:** Weight of mother and child at different stages.

	*n*	Minimum	Maximum	Mean	SD
Mother’s weight during the study	674	42.00	120.00	64.1356	11.75081
Mother’s weight before the pregnancy	671	42.00	125.00	61.8732	11.35008
Child’s age during the study	671	2.3	5.5	3.3984	1.01356
Child’s birth weight	671	1.300	4.880	3.14452	0.489787
Child’s weight in the first year	595	7.950	13.700	9.75289	1.214141
Child’s weigh at the age of two	571	9.200	18.700	12.58020	1.562586
Child’s age during the study	652	10.200	30.000	15.71304	3.028699
Mother’s age at pregnancy	674	20.0	52.0	33.393	4.5233
Mother’s age during the study	674	24	56	36.91	4.509

Notes: SD = standard deviation.

**Table 2:** Breastfeeding and BMI (Z-scores) of children for 1st year of life.

			Duration of Breastfeeding		Total
			1–6 Months	6+	
Ζ-Score 1st year	Cachectic	*n*	0	7	7
		%	0.0%	1.8%	1.2%
	Normal weight	*n*	177	325	502
		%	83.5%	84.9%	84.4%
	To the limit of becoming overweight	*n*	28	42	70
		%	13.2%	11.0%	11.8%
	Overweight	*n*	4	8	12
		%	1.9%	2.1%	2.0%
	Obese	*n*	3	1	4
		%	1.4%	0.3%	0.7%
Total		*n*	212	383	595
		%	100.0%	100.0%	100.0%
Fisher’s exact test: 7.101		*p*:	0.118	CI (99%):	0.110–0.127

**Table 3:** Breastfeeding relationship and BMI (Z-scores) of children for 2nd year.

			Duration of Breastfeeding		Total
			1–6 Months	6+	
Ζ-Score 2nd year	Cachectic	*n*	4	2	6
		%	1.9%	0.6%	1.1%
	Normal weight	*n*	163	304	467
		%	78.4%	83.7%	81.8%
	Slightly overweight	*n*	34	44	78
		%	16.3%	12.1%	13.7%
	Overweight or obese	*n*	7	13	20
		%	3.4%	3.6%	3.5%
Total		*n*	208	363	571
		%	100.0%	100.0%	100.0%
Fisher’s exact test: 4.542		*p*:	0.207	CI (99%):	0.335–0.349

**Table 4:** Relationship between breastfeeding and the Z-score of the children, taking into account the mother’s Z-score, the child’s Z-score, and the child’s gender.

ΒΜΙ (Mother)	Gender Child’s BMI			(Child) Z-Score	Duration of Breastfeeding		Total
					1–6 Months	6+	
Cachectic	Boy	Child BMI >2 to 5 years	Cachectic	*n*	1	0	1
				%	8.3%	0.0%	3.8%
			Normal weight	*n*	11	12	23
				%	91.7%	85.7%	88.5%
			Slightly overweight	*n*	0	2	2
				%	0.0%	7.7%	5.0%
	Girls	Child BMI >2 to 5 years	Cachectic	*n*	0	1	1
				%	0.0%	9.1%	5.6%
			Normal weight	*n*	7	9	16
				%	100.0%	69.2%	80.0%
			Slightly overweight	*n*	0	3	3
				%	0.0%	23.1%	15.0%
		Total		*n*	19	27	46
				%	100%	100%	100%
Normal weight	Boys	Child BMI >2 to 5 years	Normal weight	*n*	67	130	197
				%	84.8%	92.2%	89.5%
			Slightly overweight	*n*	6	9	15
				%	7.6%	6.4%	6.8%
			Overweight or obese	*n*	6	2	8
				%	7.6%	1.4%	3.6%
		Total		*n*	79	141	220
				%	100.0%	100.0%	100.0%
	Girls	Child BMI >2 to 5 years	Cachectic	*n*	2	0	2
				%	2.4%	0.0%	0.9%
			Normal weight	*n*	68	121	189
				%	81.9%	85.8%	84.4%
			Slightly overweight	*n*	10	13	23
				%	12.0%	9.2%	10.3%
			Overweight or obese	*n*	3	7	10
				%	3.6%	5.0%	4.5%
		Total		*n*	162	282	444
				%	100.0%	100.0%	100.0%
Overweight or obese	Boys	Child BMI >2 to 5 years	Cachectic	*n*	3	0	3
				%	12.0%	0.0%	4.4%
			Normal weight	*n*	17	37	54
				%	68.0%	86.0%	79.4%
			Slightly overweight	*n*	2	5	7
				%	8.0%	11.6%	10.3%
			Overweight or obese	*n*	3	1	4
				%	12.0%	2.3%	5.9%
		Total		*n*	25	43	68
				%	100.0%	100.0%	100.0%
	Girls	Child BMI >2 to 5 years	Normal weight	*n*	15	24	39
				%	78.9%	72.7%	75.0%
			Slightly overweight	*n*	3	6	9
				%	15.8%	18.2%	17.3%
			Overweight or obese	*n*	1	3	4
				%	5.3%	9.1%	7.7%
		Total		*n*	225	385	610
				%	100.0%	100.0%	100.0%

## Data Availability

The data used to support the findings of this study are available from the corresponding author upon request.
